# Influence of pH, particle size and crystal form on dissolution behaviour of engineered nanomaterials

**DOI:** 10.1007/s11356-016-7932-2

**Published:** 2016-10-26

**Authors:** M.-L. Avramescu, P. E. Rasmussen, M. Chénier, H. D. Gardner

**Affiliations:** 1 Environmental Health Science and Research Bureau, HECSB, Health Canada, 50 Colombine Driveway, Tunney’s Pasture 0803C, Ottawa, ON K1A 0K9 Canada; 2 Earth and Environmental Sciences Department, University of Ottawa, Ottawa, ON K1N 6N5 Canada

**Keywords:** Nanomaterials, Metal oxides, Zinc oxide, Titanium dioxide, ICP-MS, Dissolution, Biodurability

## Abstract

Solubility is a critical component of physicochemical characterisation of engineered nanomaterials (ENMs) and an important parameter in their risk assessments. Standard testing methodologies are needed to estimate the dissolution behaviour and biodurability (half-life) of ENMs in biological fluids. The effect of pH, particle size and crystal form on dissolution behaviour of zinc metal, ZnO and TiO_2_ was investigated using a simple 2 h solubility assay at body temperature (37 °C) and two pH conditions (1.5 and 7) to approximately frame the pH range found in human body fluids. Time series dissolution experiments were then conducted to determine rate constants and half-lives. Dissolution characteristics of investigated ENMs were compared with those of their bulk analogues for both pH conditions. Two crystal forms of TiO_2_ were considered: anatase and rutile. For all compounds studied, and at both pH conditions, the short solubility assays and the time series experiments consistently showed that biodurability of the bulk analogues was equal to or greater than biodurability of the corresponding nanomaterials. The results showed that particle size and crystal form of inorganic ENMs were important properties that influenced dissolution behaviour and biodurability. All ENMs and bulk analogues displayed significantly higher solubility at low pH than at neutral pH. In the context of classification and read-across approaches, the pH of the dissolution medium was the key parameter. The main implication is that pH and temperature should be specified in solubility testing when evaluating ENM dissolution in human body fluids, even for preliminary (tier 1) screening.

**Electronic supplementary material:**

The online version of this article (doi:10.1007/s11356-016-7932-2) contains supplementary material, which is available to authorized users.

## Introduction

Although solubility is an important parameter in risk assessments of engineered nanomaterials (ENMs), there are currently no specific standard methods for assessing the solubility of nano-objects (Arts et al. 2016; Canadian Standards Association 2011; ISO/TR 13014 2012). Water solubility and dissolution rate in simulated biological fluids are considered, along with other intrinsic material and system-dependent properties, as necessary criteria for grouping ENMs for the purpose of human health hazard assessment (Arts et al. 2014, 2015; OECD 2015). The properties of metal oxide nanomaterials (e.g. ZnO and TiO_2_) have been exploited in numerous industrial and consumer applications, including sensors, catalysts, pigments, food additives and personal care products (Fairbairn et al. 2011). Worldwide production rates are highest for nano-TiO_2_ (up to 10,000 t/year) (Piccinno et al. 2012) followed by other metal oxides including nano-ZnO (between 100 and 1000 t/year) (Ivask et al. 2012; Piccinno et al. 2012). Particle size is a key property affecting the solubility of ENMs compared to their bulk analogues (Borm et al. 2006; Misra et al. 2012) based on evidence that solubility tends to increase with decreasing particle size (Meulenkamp 1998; Mudunkotuwa et al. 2012; Schmidt and Vogelsberger 2009). Schmidt and Vogelsberger (2009) observed that TiO_2_ nanomaterials containing the amorphous form were more soluble than crystalline TiO_2_ and that pure nano-anatase was more soluble than mixed nano-anatase and nano-rutile, indicating that crystalline form can be another important factor influencing ENM solubility.

Knowledge of ENM solubility assists in interpreting potential interactions of ENMs with biological surroundings, bioavailability and persistence, uptake rates and toxicity (Arts et al. 2015; Borm et al. 2006; Cho et al. 2011; Limbach et al. 2007; Misra et al. 2012; Utembe et al. 2015). Dissolution has been identified as a critical control of biological response (Braakhuis et al. 2014; Limbach et al. 2007; Studer et al. 2010; Trouiller et al. 2009), and the observed adverse effects may be induced either by the released ions (e.g. Zn^2+^ in case of nano-ZnO) (Cho et al. 2011; Cho et al. 2012) or by persistent particles (e.g. nano-TiO_2_) (Cho et al. 2012; Limbach et al. 2007; Trouiller et al. 2009). Consequently, dissolution testing is a critical component of physicochemical characterization of nanomaterials (Arts et al. 2015; Borm et al. 2006; Studer et al. 2010; Utembe et al. 2015), and methodologies are needed to determine their dissolution rates and biodurability in biological fluids.

Biological fluids are difficult to simulate, however, and the greater the complexity of constituents in simulated body fluids, the greater the challenge in measuring and describing nanomaterial dissolution (Kittler et al. 2010). A variety of different constituents, pH and temperature regimes, which vary depending on the purpose of each study, have been used to assess solubility of nanomaterials. Examples include the following: phosphate buffer saline (PBS) adjusted to pH 1.2, 6.8 and 7.4 to simulate gastric, intestinal and plasma conditions respectively (Gwak et al. 2015); NaCl/pepsin solution adjusted to pH 1.5 with HCL to simulate gastric conditions (Cho et al. 2013); Dulbecco’s modified Eagle’s medium (DMEM), pH 7.68 and BEGM held at 37 °C (Mu et al. 2014; Xia et al. 2008); ALF and Gamble solution at 38 °C to simulate alveolar and interstitial lung fluid respectively (Stebounova et al. 2011); 0.01 M Ca(NO_3_)_2_ buffered with 2 mM piperazine-*N*,*N*′-bisethanesulfonic acid (PIPES) to pH 7.5 ± 0.1 held at 21 °C (Yin et al. 2015). Constituents of simulated biological media, such as organic and inorganic ligands, may either increase or decrease dissolution, and therefore, the impact of media components on solubility must be understood for each ENM (David et al. 2012; Li et al. 2011; Mu et al. 2014; Mudunkotuwa et al. 2012).

The present study addresses the need for information on water solubility of ENMs for preliminary categorization and “tier 1” hazard assessments, as described by Arts et al. (2015). At present, no standardized protocol exists for determination of ENM water solubility (Arts et al. 2016). As pH and temperature of the medium are key parameters influencing dissolution of metals assessed using in vitro assays (Koch et al. 2013, Stefaniak et al. 2005), this study assessed dissolution of ENMs at body temperature (37 °C) using two pH conditions (1.5 and 7) to approximately frame the pH range found in human body fluids. The solubilities of Zn metal, ZnO and two TiO_2_ ENMs were compared to their bulk analogues on the basis of a 2-h assay, typical of traditional metal bioaccessibility assays (Koch et al. 2013; Dodd et al. 2013). To minimize any inadvertent effects of media composition on ENM dissolution (for the purpose of preliminary water solubility assessments), simple components were added solely for the purpose of adjusting pH (0.07 M HCl for low pH and 0.01 M ammonium acetate for neutral pH). The present study also addresses the recommendation by Utembe et al. (2015) to determine dissolution rate constants in order to understand biodurability. Time series experiments were conducted at body temperature and two pH conditions to calculate rate constants and half-lives of ZnO and TiO_2_ nanomaterials and their bulk analogues. The results were used to assess the influence of particle size, crystal form and pH on dissolution behaviour of the investigated materials.

## Materials and methods

### Nano-powders, bulk powders and reagents

Uncoated ZnO, TiO_2_ (anatase and rutile) and Zn metal nanomaterials and their bulk analogues were purchased from Sigma-Aldrich (Oakville, ON, Canada) and Alfa Aesar (Ward Hill, MA, USA). NIST 1898 TiO_2_ (anatase and rutile mixture) Standard Reference Material (SRM) was purchased from the National Institute of Standards and Technology (Gaithersburg, MD, USA). Characteristics of the test materials are summarized in Table 1. Zinc chloride (ZnCl_2_) and ammonium acetate were obtained from Sigma-Aldrich, and high purity hydrochloric, nitric and hydrofluoric acids were obtained from SEASTAR Chemicals Inc. (Sidney, BC, Canada). Certified reference materials for trace element quality control (low- and high-level fortified waters TM-28.4 and TMDA 54.5 respectively) were purchased from Environment Canada (Ottawa, ON). Ti and Zn calibration standards and Ge internal standard were prepared using high purity standard stock solutions (1000 μg/mL; Delta Scientific Ltd.; Mississauga, ON, Canada). Ultrapure Milli-Q water (18.2 MΩ cm) was used for preparation of all reagents and calibration standards.

**Table 1:** Summary of physical–chemical characteristics of materials used in this study as reported by manufacturer and measured using XRD and SAXS (details in Supplementary Material)

Sample ID	Reported by manufacturer			XRD and SAXS results			
	Particle size	SSA (m^2^/g)	Density (g/cm^3^)	Rietveld analysis (%)	Diameter (nm) by SAXS	Scherrer diameter^a^ (nm)	Calc. SSA^b^ (m^2^/g)
Nano-ZnO_50 nm_	<50 nm	>10.8	5.61		36	33	32.4
Nano-ZnO_100 nm_	<100 nm	15–25	5.61			48	22.3
Bulk-ZnO	Not reported		5.61			85	12.6
Nano-Zn metal	<50 nm^c^	35–50	7.133				
Bulk-Zn metal	∼100 mesh						
Nano-rutile (TiO_2_)	<100 nm	50	4.17	98 % rutile		80	18.0
				2 % anatase		81	
Bulk-rutile (TiO_2_)	<5 μm		4.17	97 % rutile		123	11.7
				3 % anatase		99	
Nano-anatase (TiO_2_)	<25 nm	45–55	3.9	100 % anatase	10	7	154
Bulk-anatase (TiO_2_)	Not reported		3.9	100 % anatase		68	22.6
NIST 1898 (TiO_2_)	37 ± 6 nm (rutile)	55.55 ± 0.70		13 % rutile	29	35	41.1
(24 % rutile and 76 % anatase)	19 ± 2 nm (anatase)			87 % anatase		19	81.0

^a^Greater uncertainty outside optimum range for Scherrer (1–65 nm, potentially extending to 100 nm)

^b^Calculated specific surface area assuming spherical particles of uniform size: SSA = 6/*ρ*/*d* (d = Scherrer diameter; *ρ* = density)

^c^Manufacturer note that after sonication the material is disagglomerated into its primary nanoparticles of about 35 nm

### Characteristics of nanomaterials and analogues used in the study

Powdered X-ray diffraction (XRD) and small-angle X-ray scattering (SAXS) was conducted using a Rigaku Ultima IV Diffractometer (University of Ottawa X-ray facility) to determine crystallographic structure and confirm purity. Detailed information about the XRD and SAXS methods and results are provided in the Supplementary Material. The Scherrer nanocrystal diameter estimates for all ZnO and TiO_2_ samples are summarized in Table 1, along with information provided by the manufacturers. Generally, the Scherrer calculations for nanocrystal diameter and SAXS estimates for nanoparticle size agreed with the manufacturer specifications. Powdered XRD identified all ZnO samples used in this study as wurtzite (Fig. S1, Supplementary Material). With respect to TiO_2_ crystal forms, the powdered diffraction patterns for both nano- and bulk-anatase samples identified anatase as the only form present. However, the nano- and bulk-rutile powders all contained anatase as a minor constituent (less than 4 % by Rietveld analysis; Table 1). Sample purity with respect to trace metal contaminants was confirmed using ICP-MS/ ICP-OES microwave-assisted acid digestion.

### Instrumentation and QA/QC

Samples were weighed with a Mettler Toledo XP205 digital analytical balance equipped with a U-shaped anti-static electrode. When handling the ENMs, appropriate personal protective equipment was worn (face mask and gloves) and care was taken to avoid inadvertent generation of aerosols. The Hach 40d pH meter used to monitor the pH during experiments was calibrated daily using three different buffer solutions (pHs 4, 7, 10).

Metal concentrations were determined using either the NexION 300s Dual-channel Universal Cell ICP-MS (Perkin Elmer, Canada) or the Optima 5300V ICP-OES (Perkin Elmer, Canada). The ICP-MS was equipped with a SC-Fast autosampler (Elemental Scientific, Omaha, NE), a high-temperature apex-ST PFA MicroFlow nebulizer, cyclonic spray chamber with PC3x chiller (2 °C) and a triple cone interface (nickel–platinum skimmer and sampler cone, and aluminium hyper cone). The following conditions were used: argon flow rates of 18, 1.2 and 0.9–1 L/min for plasma, auxiliary and nebulizer respectively, and 1600 W forward RF power. Optimization was carried out daily with a normal tuning solution (1 ng/mL Be, Ce, Fe, In, Li, Mg, Pb, U). Three replicate readings were taken for all monitored masses and elements. The Optima 5300V ICP-OES equipped with radial optical system (163 to 782 nm range) was used at the wavelengths recommended by the manufacturer. The instrument was operated at 1400 W power and flow rates of 15, 0.2 and 0.9 L/min for plasma, auxiliary and nebulizer respectively. Daily instrument tuning was performed using a solution of 10 mg/L Mn (2 % HNO_3_).

Microwave-assisted acid digestions (for total metal determinations) were performed using the Ethos Touch Control Advanced Microwave Labstation (Milestone Microwave Laboratory Systems) equipped with Ethos TC built-in ATC-400-CE automatic temperature control. The following temperature program was used: 20 min to reach 180 °C, ramp from 180 to 220 °C (10 min, 1000 W) followed by 20 min at 220 °C (1000 W).

Procedural blanks consisting of extraction media (same reagent mixture and dilution factors as the samples) were run with each batch to evaluate inadvertent contamination of samples and to calculate matrix blank corrections where appropriate. Detection limits determined from procedural blanks were: 60 ng Zn/L and 77 ng Ti/L (low pH) and 78 ng Zn/L and 19 ng Ti/L (neutral pH) by ICP-MS, and 42 μg Zn/L (low pH) and 47 μg Zn/L (neutral pH) by ICP-OES. Spiked media samples consisting of ZnCl_2_ (0.4 mg/mL Zn) and Ti (0.3 mg/mL Ti) were run in triplicate with each batch to test for metals loss in the tube walls, retained on filter and/or precipitation. Recoveries obtained for the spiked media samples were in the range of 91–102 % for Zn and 91–106 % for Ti with both low and neutral pH extractions. No difference was observed for Zn and Ti spike recoveries between the beginning and the end of time series experiments at both pHs (RSDs in the range of 0.2–3 % for Zn and 0.4–0.9 % for Ti) which indicated that dissolved Zn and Ti were neither retained on filter or tube walls nor leached from the filter material. Evaluation of TM-28.4 and TMDA 54.5 reference materials indicated recoveries in the range of 93–112 % for both Zn and Ti. The pH of the extracts, monitored before and after each experiment, fell within the range of 1.3 to 1.5 (gastric fluid) and within the range of 6.8 to 7.5 for neutral pH assays.

### Experimental parameters used for dissolution assays

All dissolution experiments were conducted using low pH (1.5) and neutral pH (7) solutions maintained at 37 °C (body temperature). The low pH assay used a weak (0.07 M) HCl solution to simulate gastric conditions as optimized by Rasmussen et al. (2008). The neutral pH assay used a 0.01 M ammonium acetate (AA) solution based on a method by Thomassen et al. (2001) to simulate the neutral lung environment in an occupational study and later applied to incidental nanoparticles by Niu et al. (2010). The key modification to these methods was the introduction of syringe filtration using a 0.02-μm filter (Anotop 25 mm; Whatman) to separate the dissolved fraction (*M*_d_) from undissolved particles. This modification was based on a comparison of syringe vs centrifugation approaches which demonstrated that the original separation method (centrifugation at 3500 rpm) did not adequately separate particulate matter, evidenced by erroneously elevated apparent *M*_d_ values. The comparison of syringe filtrate and centrifugation values for the short 2-h assays is available in the Supplementary Material (Table S1). Although centrifugal ultrafiltration (<3-kDa ultrafilters, Amicon Ultra; Millipore) compared well with the syringe filtration results, the time delay created by the required 30 min centrifugation step was not compatible with the time series experiments used in this study.

### Short solubility assays

Short (2 h) assays were designed to enable calculation of solubility of the test ENMs and their bulk analogues in terms of concentration units (mg/L or μg/L) and as percentage (%) of metal dissolved from the total original metal concentration (dissolved/original material [*M*_d_/*M*_o_]). The short assays used a 25-mg test sample in a 50-mL polypropylene centrifuge tube with a 50 mL aliquot of extraction media (either low gastric pH or neutral lung pH). The extraction tubes were placed in a covered shaker hot water bath (37 °C) for 2 h (1 h with agitation followed by 1 h without agitation). The pH levels were monitored before, during and after each experiment using a Hach 40d pH meter.

To prevent the contamination of the test suspensions, the samples were poured directly into the syringes with the filter installed (Reed et al. 2012). After separation, all extracts were acidified with HNO_3_ to a final concentration of 3 % and diluted as required prior to ICP-OES and/or ICP-MS analyses. Five procedural blanks and spiked matrix blanks (Ti or Zn) were run with each batch for calculation of mean and standard deviation. Appropriate matrix blank correction was applied for all experimental runs.

### Time series experiments to calculate dissolution kinetics

Time series experiments were designed to permit calculation of dissolution rates using the same method described above, with the exception that shaking in the 37 °C bath was maintained continuously over a longer dissolution time. Triplicates of each sample were dispersed in the appropriate media (low pH and neutral pH) at an initial concentration of 0.5 mg/mL metal oxide, which is equivalent to that of the short assay. Aliquots were collected from each replicate suspension at the following times: immediately after mixing, at 10, 20, 30, 60, 120, 180 and 240 min (also at 24 h for TiO_2_ samples at neutral pH). The solid component was removed immediately by syringe filtration (0.02-μm filter, Anotop 25) and the filtrate was acidified to 3 % nitric acid. Concentrations of dissolved metal (*M*_d_) were determined by ICP-MS or ICP-OES. In addition, aliquots of 1 mL suspension were collected at the beginning and at the end of each experiment to confirm the total metal concentration (using microwave digestion and ICP-MS). For all dissolution experiments, the mean and standard deviation were reported.

The rate of dissolution was calculated for ZnO samples at low pH according to a modified first-order equation (Eq. 1) using the mass fraction of dissolved Zn/original compound (*M*_d_/*M*_o_):

1 $$ {y}_t = {y}_{\mathrm{final}} \times \left(1-{e}^{-kt}\right) $$

where *y*_*t*_ = the mass fraction of dissolved Zn/original material (*M*_d_/*M*_o_) at time *t* (days); *M*_o_ = the original mass of material (Zn); *M*_d_ = the mass fraction of the dissolved material (Zn); *y*_final_ = the degree of dissolution; and *k* (day^−1^) = the dissolution rate constant expressed in days.

The mass fractions of the dissolved/original material (*M*_d_/*M*_o_) were plotted as a function of time, and suitable non-linear regression models were fitted to extract the values of *k* and *y*_final_. This enabled calculation of the half-life (*t*_1/2_ = ln(2) / *k*) which corresponds to the time to reach *y*_final_/2. This approach has been used previously to quantify dissolution of ENMs (Kittler et al. 2010; Majedi et al. 2014) and corresponds to a product formation of a first order reaction normalized to *y*_final_.

It was difficult to calculate a half-life using Eq. 1 for TiO_2_ compounds and ZnO at neutral pH because the degree of dissolution was so small (*y*_final_ < 0.5; as observed by Kittler et al. 2010 for nano-Ag). For these cases, it was necessary to derive the half-life using the ratio of material remaining/original material (*M*_r_/*M*_o_) instead of material dissolved/original material (*M*_d_/*M*_o_). This was done using Eq. 2, which incorporated the size dependence of dissolution (surface area-normalized rate law) (Mercer 1967):

2 $$ y=f\times {e}^{-kt} $$

where *y* = the mass fractions of the remained/original material (*M*_r_/*M*_o_) at time *t* (days); *M*_o_ = the original mass of material (Zn or Ti); *M*_r_ = the mass fraction of the remaining material (*M*_r_ = *M*_o_ − *M*_d_); *f* = fraction of original material dissolved; and *k* (day^−1^) = the rate constant (in days) that, normalized to the specific surface area (SSA, cm^2^/g), provides the surface area-normalized dissolution rate constant (k_SSA_ = *k*/SSA, g/(cm^2^ day^−1^)).

For bulk-ZnO dissolution at neutral pH, the time series data were better described by Eq. 2a, which also uses the remaining material (*M*_r_) in the mass fraction, but is a biphasic model with two negative exponential functions (instead of one negative exponential as in Eq. 2):

2a $$ y={\displaystyle \sum_{i=1}^n}{f}_i{e}^{-{k}_it} $$

where *f*_*i*_ = the fraction of total material dissolved in each phase ($\sum_{i=1}^n{f}_i=100\%\Big)$ and corresponds to the percent of material available for absorption per phase (Stefaniak et al. 2010). The dissolution of bulk-ZnO in this time series was well described by two negative exponentials (*r* ^2^ = 0.996, *p* < 0.0001), which correspond to biphasic dissolution with a rapid initial phase followed by a longer-term phase.

In summary, the mass fractions (either dissolved/original or remaining/original) for each time series experiment were plotted as a function of time. Non-linear regression models with one (single dissolution phase) or two (biphasic) component negative exponentials were fitted. When necessary, the number of components (*i*) was selected using the *F*-ratio test. The outputs of the chosen model were then used to calculate each sample surface area-normalized first-order dissolution rate constant (k_SSA_ = *k*/SSA) and half-life *t*_1/2_ = ln(2)/*k* (Mercer 1967; Stefaniak et al. 2010).

Sigma Plot statistical software (v. 13.0.0.83) was used for Student’s *t* test and Mann–Whitney Rank Sum test as required for comparisons of two sample sets and ANOVA for multiple sets (Holm–Sidak method for specific differences of means).

## Results and discussion

**Fig. 1:**
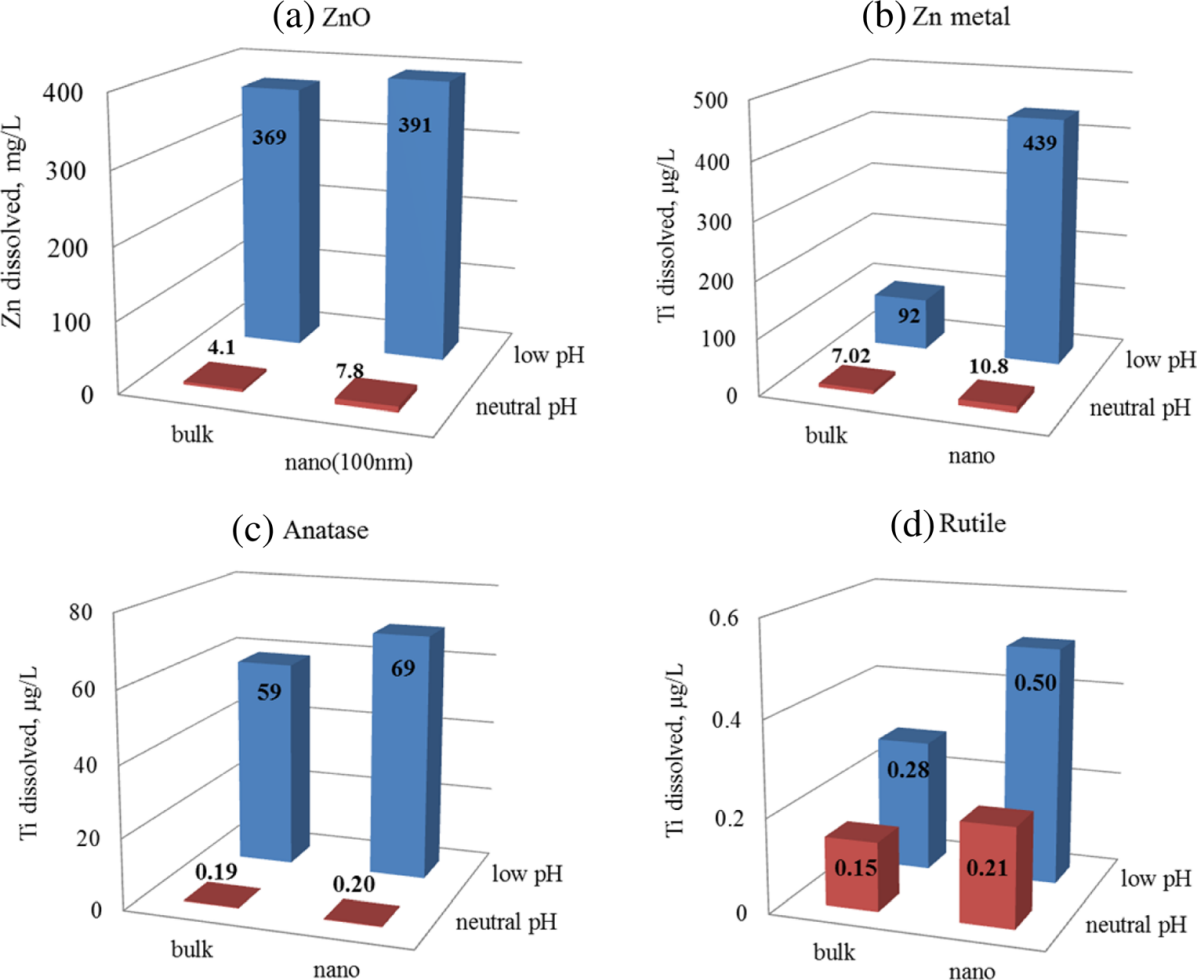
Concentration of **a**, **b** Zn (mg/L) and **c**, **d** Ti (μg/L) released from nano- and bulk-ZnO, Zn metal and TiO_2_ materials estimated with short (2 h) solubility assay (syringe filtration) at low and neutral pH. Results are presented as mean and standard deviation of their individual replicates

**Fig. 2:**
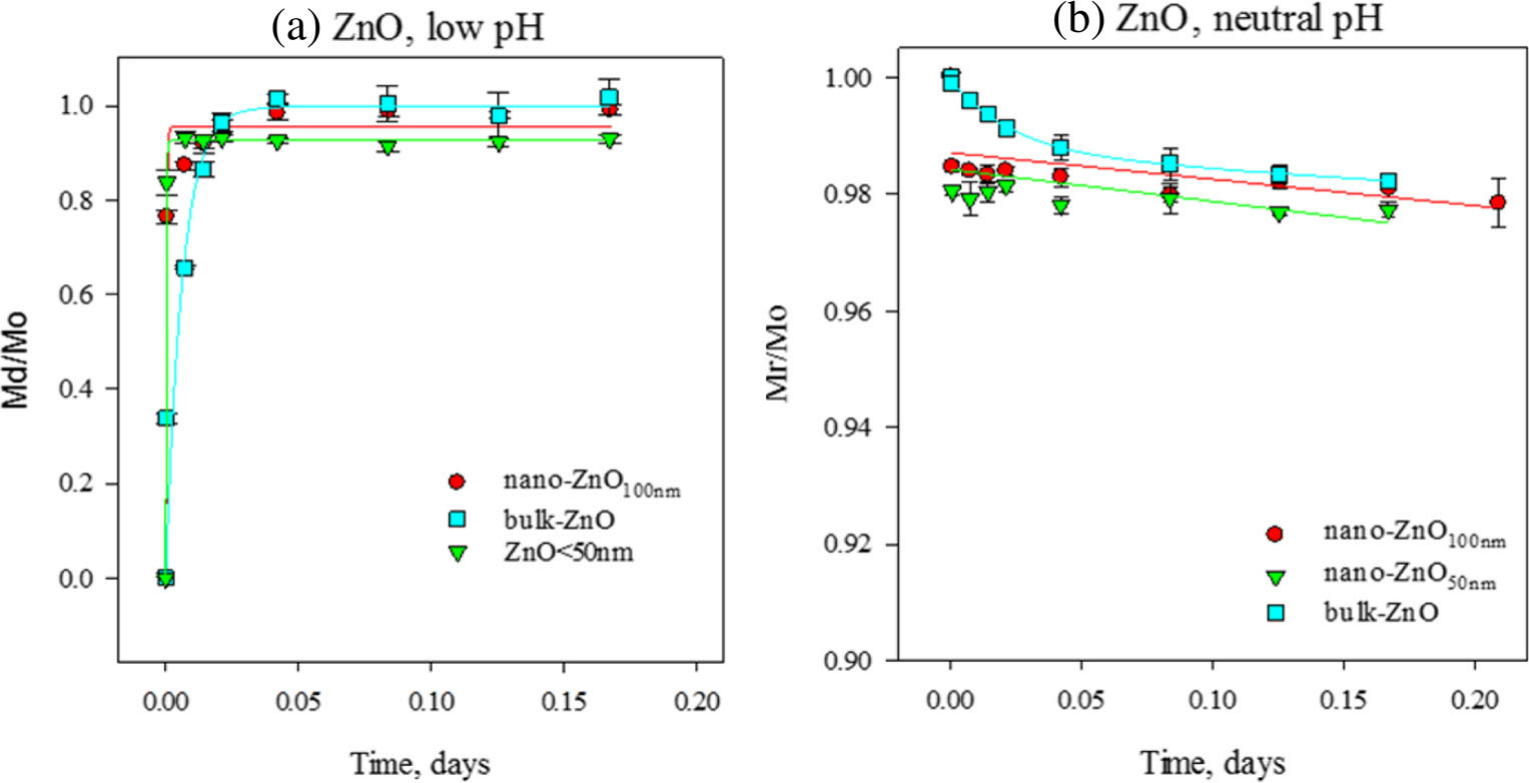
Dissolution kinetics for ZnO nanomaterials and bulk analogues at **a** low pH and **b** neutral pH. **a** Mass fractions of the dissolved/original material (*M*_d_/*M*_o_) fitted using Eq. 1 (see text). **b** Mass fractions of the remaining/original material (*M*_r_/*M*_o_) fitted using Eq. 2 (Eq. 2a for bulk-ZnO as described in text). *Error bars* represent standard deviations of three individual replicates

**Fig. 3:**
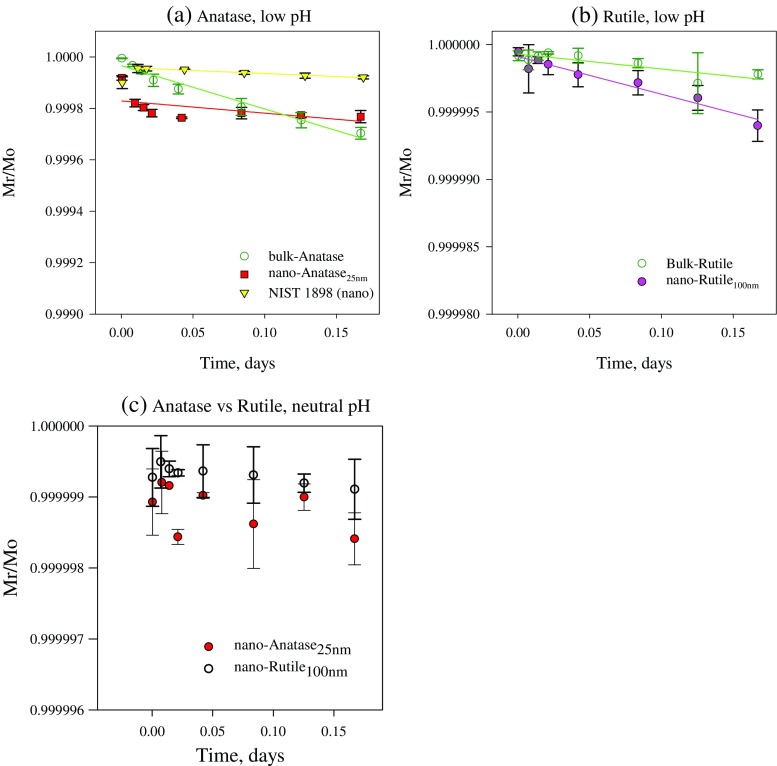
Dissolution kinetics of anatase (**a**) and rutile (**b**) from nanomaterials and bulk analogues at low pH (**a**, **b**). The mass fractions of the material remained/original material (*M*_r_/*M*_o_) titanium were fitted with Eq. 2. **c** The average of the remaining/original material (*M*_r_/*M*_o,_ *n* = 3) vs time for both TiO_2_ NMs (anatase and rutile) at neutral pH. Anatase dissolution is significantly higher than rutile (Mann–Whitney Rask Sum Test *p* = 0.0006 for pairs). *Error bars* represent standard deviations of three individual replicates

Results of the simple 2 h solubility assay are presented first (Table 2; Fig. 1), followed by the time series experimental results which are used to calculate dissolution rate constants and half-lives (Table 3; Figs. 2 and 3). The dissolution characteristics of ZnO, zinc metal and TiO_2_ nanomaterials are compared with those of their bulk analogues for both pH conditions evaluated. Two crystal forms of TiO_2_ are considered: anatase and rutile.

### Solubility expressed as concentration units and percentages (2-h assay)

For the purpose of classification for risk assessment, ENM solubility may be expressed in concentrations units (e.g. mg/L or μg/L), as in the classification scheme presented by Arts et al. (2015), or as percentages (% dissolved mass/total mass), as in the classification scheme presented by OECD (2015). The histograms in Fig. 1 compare solubility of ENMs and their bulk analogues using concentrations of Zn (mg/L) and Ti (μg/L) released during the 2-h solubility assay at low and neutral pH. These results are provided in Table 2 as both concentrations and percentages.

**Table 2:** Dissolution results presented as concentration (mg/L or μg/L) and percent dissolved of nano- and bulk-ZnO and TiO_2_ samples at low pH (1.5) and neutral pH (pH 7) obtained with the short (2 h) assay (syringe filtration)

Description	Bulk	Nano	Bulk *vs* nano^b^
Neutral pH			
Zn metal	7.02 ± 0.39 mg/L or 1.33 %	10.8 ± 0.50 mg/L or 2.05 % (<50 nm)	*p < 0.001*
ZnO	4.12 ± 0.21 mg/L or 0.95 %	7.85 ± 0.27 mg/L or 1.87 % (<100 nm)	*p < 0.0001*
		8.59 ± 0.45 mg/L or 2.13 % (<50 nm)	*p < 0.0001*
Rutile	0.146 ± 0.069 μg/L or 0.000048 %	0.210 ± 0.058 μg/L or 0.000066 % (<100 nm)	*p = 0.408*
Anatase	0.190 ± 0.075 μg/L or 0.000059 %	0.197 ± 0.069 μg/L or 0.000065 % (<25nm)	*p = 0.242*
Nist 1898^a^		0.128 ± 0.040 μg/L or 0.000041 %	
Low pH			
Zn metal	92.3 ± 21.4 mg/L or 17.5 %	439.2 ± 24.4 mg/L or 80.8 % (<50 nm)	*p < 0.0001*
ZnO	368.7 ± 15.3 mg/L or 88.5 %	391.3 ± 5.11 mg/L or 97.0 % (<100 nm)	*p = 0.0098*
		379 ± 5.25 mg/L or 93.6 % (<50 nm)	*p = 0.128*
Rutile	0.282 ± 0.032 μg/L or 0.000092 %	0.505 ± 0.059 μg/L or 0.00016 % (<100 nm)	*p < 0.0001*
Anatase	58.5 ± 6.08 μg/L or 0.019 %	69.3 ± 6.44 μg/L or 0.022 % (<25 nm)	*p = 0.019*
Nist 1898		39.1 ± 3.65 μg/L or 0.012 %	

Data are presented as mean and standard deviation of five independent replicates

^a^ *n* = 3; ^b^results for bulk *vs *nano are significantly different when ﻿﻿*p* < 0.05

Table 2 shows that, using the short 2-h assay, nano-Zn metal displayed significantly higher solubility (*p* < 0.001) than its bulk analogue at both low and neutral pH conditions. At neutral pH, both ZnO nanomaterials displayed significantly higher solubilities (*p* < 0.001) than their bulk-ZnO analogue (Table 2), with the solubility of ZnO_50 nm_ being significantly higher (*p* = 0.003) than that of ZnO_100 nm_. At low pH, the solubility of both ZnO nanomaterials was also higher than the ZnO bulk analogue, although the difference was significant only for ZnO_100 nm_ (Table 2). At low pH, solubility was significantly higher (*p* < 0.05) for both nano-anatase and nano-rutile compared with their bulk analogues. At neutral pH, the solubilities of nano-anatase and nano-rutile were slightly higher than their bulk-TiO_2_ analogues, but the difference was not statistically significant (Table 2).

The histograms (Fig. 1) contrast the solubilities of the test materials at low pH and neutral pH using the 2-h assay. All nanomaterials displayed significantly higher solubility at low pH than at neutral pH (*p* < 0.01 for nano-ZnO and nano-Zn metal; *p* < 0.001 for nano-rutile; *p* = 0.016 for nano-anatase). The differences were even more significant for their bulk analogues (*p* < 0.0001 for ZnO and Zn metal; *p* < 0.01 for anatase and rutile). These results confirm that the solubility of metal compounds (regardless of particle size) is strongly pH dependent.

The 2-h assay results presented in Table 2 show that the solubility of TiO_2_ ENMs and their bulk analogues was very low (from 0.15 to 69.3 μg/L or 3.1 nmol/L to 1.4 μmol/L) within the pH values tested. This is in agreement with the range obtained by Schmidt and Vogelsberger (2009) for nano-TiO_2_ (1 nmol/L to 2 μmol/L), which varied depending on the pH and temperature of the medium used (Schmidt and Vogelsberger 2006, 2009). Dissolution studies of TiO_2_ compounds are scarce as they are generally assumed to be insoluble, and there are also analytical challenges in measuring low concentrations of dissolved Ti, which require high sensitivity and special care to minimize contamination (Schmidt and Vogelsberger 2006, 2009).

At low pH, the solubility of nano-anatase was observed to be significantly higher (*p* = 0.016) than that of nano-rutile (Table 2; Fig. 1c, d). The influence of crystal form on solubility was also observed for the bulk anatase and rutile samples at low pH (Fig. 1c, d). These results are consistent with thermodynamic studies of TiO_2_ (Lencka and Riman 1993) and are relevant to cytotoxicity studies which found that nano-anatase is about 100 times more toxic than nano-rutile (Sayes et al. 2006). The solubility of NIST 1898 was significantly lower (*p* < 0.001) than nano-anatase and significantly higher (*p* < 0.01) than nano-rutile (Table 2), as expected, since this SRM is a mixture of crystal forms (76 % nano-anatase and 24 % nano-rutile).

This section showed the effect of particle size, pH and crystallinity on the solubility of Zn metal, ZnO and TiO_2_ and investigated the capabilities of a simple 2-h assay to distinguish between nanomaterials and their bulk analogues. The next section investigates how these same properties of ZnO and TiO_2_ compounds impact dissolution kinetics under the same set of pH and temperature conditions.

### Solubility expressed using dissolution rate constants and half-lives

This section presents the results of the time series dissolution experiments and the calculation of dissolution rate constants and half-lives for the ZnO and TiO_2_ nanomaterials and their bulk analogues. Table 3 summarizes the dissolution parameters (mean ± SE, *n* = 3) of ZnO materials at both pH conditions and TiO_2_ materials at low pH. Equation 2 was used for all cases in Table 3 except ZnO materials at low pH where Eq. 1 was used.

**Table 3:** Dissolution parameters (mean ± SE, *n* = 3) of ZnO materials at low pH conditions (pH 1.5) and neutral pH (pH 7) and TiO_2_ materials at low pH conditions (pH 1.5)

Sample ID	pH	*f* (%)	*k* (day^−1^)	*t*_1/2_ (day^−1^)	*k* (g/cm^2^/day)	*r* ^2^	*p* value
Nano-ZnO_50 nm_	1.5	92.6 ± 0.26	5320 ± 186	1.30 × 10^−4^	1.64 × 10^−2^	0.998	<0.0001
Nano-ZnO_100 nm_	1.5	95.6 ± 0.90	3275 ± 251	2.12 × 10^−4^	1.31 × 10^−2^	0.984	<0.0001
Bulk-ZnO	1.5	99.8 ± 2.76	159 ± 24.1	43.7 × 10^−4^	0.126 × 10^−2^	0.919	<0.0001
Nano-ZnO_50 nm_	7	98.4 ± 0.20	0.056 ± 0.022	12.5	1.73 × 10^−7^	0.213	0.0154
Nano-ZnO_100 nm_	7	98.7 ± 0.10	0.046 ± 0.013	15.8	1.85 × 10^−7^	0.314	0.0016
Bulk-ZnO^a^	7	98.7 ± 0.20	0.030 ± 0.014	23.1	2.38 × 10^−7^	0.969	<0.0001
Nano-anatase^b^	1.5	99.988 ± 0.001	3.26 ± 0.61 × 10^−3^	213	5.92 × 10^−9^	0.687	0.0001
Bulk-anatase	1.5	99.997 ± 0.0008	1.68 ± 0.09 × 10^−3^	412	7.44 × 10^−9^	0.936	<0.0001
Nano-rutile	1.5	100 ± 0.00	2.79 ± 0.33 × 10^−5^	24,836	1.55 × 10^−10^	0.763	<0.0001
Bulk-rutile	1.5	100 ± 0.00	1.11 ± 0.30 × 10^−5^	62,608	0.94 × 10^−10^	0.376	0.0014
NIST 1898	1.5	99.996 ± 0.0003	0.23 ± 0.03 × 10^−3^	2954	4.22 × 10^−10^	0.756	<0.0001

Parameters were estimated with Eq. 2 except for ZnO materials at low pH where Eq. 1 was used

*SE* standard error of the regression coefficient

^a^Data described by two negative exponentials corresponds to biphasic dissolution behaviour. The fraction of the bulk-ZnO dissolved in the initial phase was less than 2 % (*f* = 1.24 ± 0.20 %, *k* = 42.9 ± 10^−4^ day^−1^; *t*_1/2_ = 0.016 day and k_SSA_ = 3.41 × 10^−4^ g/cm^2^/day), and consequently, the reported dissolution rate corresponds to the long-term phase

^b^First 60 min data fitted (parameters from all data fitted model: *k* = 0.47 ± 0.09 × 10^−3^ day^−1^, *t*_1/2_ = 14.6 × 10^−2^ day, k_SSA_ = 0.86 × 10^−9^ g/cm^2^/day, *r* ^2^ = 0.34 and *p* = 0.15. See supplementary material for explanation)

#### Dissolution kinetics of ZnO compounds

The difference between the two approaches to describe dissolution kinetics mathematically is illustrated in Fig. 2 for ZnO nanomaterials and their bulk analogues at low pH (1.5) and neutral pH (7). Equation 1, which is used in Fig. 2a, describes the dissolved fraction (sometimes called the “bioaccessible” fraction) and is useful for calculating half-life when there is a sufficient concentration of dissolved metal (i.e. more than half the original mass has dissolved). Because the dissolution rates of ZnO nanomaterials and their bulk analogue at low pH were very fast (apparent equilibrium reached within 30 min), this dataset was best described by Eq. 1 (Fig. 2a). The degree of dissolution observed at the end of the time series (92.9 to 100.3 %) matched that calculated from the Eq. 1 model (92.6 to 99.8 %), supporting the capability of using Eq. 1 to describe these experimental results (Eq. 1 does not apply to the neutral pH dataset, because a true half-life could not be defined due to the low degree of dissolution).

Equation 2, which is used in Fig. 2b, describes the undissolved fraction and is therefore useful for quantifying biodurability (also called “biopersistence”) of less soluble materials (where less than half the original mass has dissolved). At neutral pH, the dissolution rate of ZnO materials was slow, with an apparent equilibrium reached within about 60 min. This slow dissolution was best described by Eq. 2 using the remaining/original mass fraction as shown in Fig. 2b. Consequently, Eq. 2 was used to extract *k* values and calculate half-lives of all the ZnO materials at neutral pH as presented in Table 3 (Eq. 2 does not apply to the low pH dataset, due to the rapid dissolution of ZnO at low pH).

The trends in dissolution kinetics for ZnO compounds (Table 3) correspond with those of the 2 h solubility assay (Fig. 1; Table 2). Both ZnO nanomaterials tested displayed higher *k* values (dissolution rate constants) and k_SSA_ values (rate constants normalized using specific surface area) and corresponding shorter half-lives than their bulk analogue (Table 3). At both evaluated pH conditions, the dissolution (rates) of ZnO materials increased (and half-lives decreased) with decreasing particle size in the following order: bulk-ZnO < nano-ZnO_100 nm_ < nano-ZnO_50 nm_, consistent with the results obtained with the 2-h solubility assay at neutral pH (Table 2). Results of the 2-h assay were less consistent than the time series results for nano-ZnO_50 nm_ at low pH (Table 2). Schmidt and Vogelsberger (2009) also observed such inconsistencies in ENM solubility at low pH, which they attributed to variability in the quality of commercial products (from one manufacturer to another).

The dissolution kinetics of ZnO compounds also correspond with the 2 h solubility assay results with respect to the effect of pH, in that dissolution rates of both ZnO nanomaterials were significantly slower in neutral pH media than low pH media. For example Table 3 shows that for ZnO_50 nm_, *k* = 5320 day^−1^ at low pH compared to *k* = 0.056 day^−1^ at neutral pH. This translates into shorter half-lives at low pH than at neutral pH (e.g. *t*_1/2_ = 11 s at low pH and *t*_1/2_ = 12.5 day at neutral pH for ZnO_50 nm_).

#### Dissolution kinetics of TiO_2_ compounds

The results of the time series experiments confirmed that crystal form is an important characteristic that influences TiO_2_ solubility. All TiO_2_ materials dissolved very slowly and reached very low dissolved concentrations at both pHs (Fig. 3; Table 3). However, at low pH, the half-life of nano-anatase was 116 times shorter than the half-life of nano-rutile, with correspondingly higher *k* and k_SSA_ values (Table 3). At neutral pH, changes in *M*_r_/*M*_o_ over time were too subtle to fit a curve for each compound, but differences in dissolution behaviour amongst the compounds could be evaluated by comparing their respective *M*_r_/*M*_o_ values. The greater solubility of nano-anatase compared to nano-rutile at neutral pH (Fig. 3c) was evident at the end of the time series (240 min) when the *M*_r_/*M*_o_ fraction for nano-rutile was 1.8 times higher (*p* < 0.05) than that of nano-anatase (in contrast to the nanomaterials, no difference could be discerned between the dissolution of bulk-rutile vs bulk-anatase at neutral pH). The reference TiO_2_ nanomaterial NIST 1898 demonstrated dissolution behaviour between that of nano-anatase and nano-rutile (Table 3), as observed in the 2-h solubility assay, with a higher dissolution rate constant (*k* = 0.23 × 10^−3^ day^−1^) and a lower half-life (*t*_1/2_ = 2954 day) than nano-rutile (*k* = 2.79 × 10^−5^ day^−1^; *t*_1/2_ = 24,836 day; Table 3).

Evaluation of the *M*_r._/*M*_o_ fraction at the end of the time series was also useful for comparison of the effect of pH on dissolution of the test materials. Similar to the results of the above 2-h assay, significantly more material remained undissolved at neutral pH than at low pH for all TiO_2_ compounds (nano-anatase *p* < 0.0001; nano-rutile *p* = 0.001; bulk rutile *p* = 0.0002; and bulk anatase *p* = 0.0002).

At low pH, both TiO_2_ nanomaterials tested displayed higher k values (dissolution rate constants) and k_SSA_ values (rate constants normalized using specific surface area) and corresponding shorter half-lives than their bulk analogues (Table 3). In fact, the bulk rutile half-life (6.26 × 10^4^ days) was more than double that of nano-rutile (2.48 × 10^4^ days). At neutral pH, no difference could be discerned between the dissolution of TiO_2_ nanomaterials and their bulk analogues in the time series experiments (*p* = 0.063; not shown).

### Comparison of nanomaterials with their bulk analogues

The term “read-across” refers to the use of test results for a non-nanoscale material (the “bulk” material in the present study) to predict the behaviour of its analogous nanomaterial in the absence of test results for that nanomaterial (Arts et al. 2015, 2016; Patlewicz et al. 2013). It would be advantageous if risk assessors, risk managers and regulators could rely on aqueous solubility information from standard references such the *CRC Handbook of Physics and Chemistry* (Haynes 2015) in the absence of nanomaterial-specific solubility information as a decision support tool. However, the results of this study demonstrated the importance of specifying the pH of the medium when grouping or classifying ENMs according to water solubility.

The importance of pH is illustrated by Table 4, which places the results of the short (2 h) assays into the context of solubility categories based on concentration units and percentages (%) of metal dissolved from the total original metal concentration (*M*_d_/*M*_o_). Using the tier 1 screening criterion of 100 mg/L suggested by Arts et al. (2015), Table 4a shows that nano-Zn metal and all ZnO compounds, regardless of particle size, classified as “soluble” at low pH and “biopersistent” at neutral pH. Table 4b shows that similar groupings arise based on percentage solubility screening criteria from OECD (2015). The advantage of dividing solubility into four categories (as in Table 4b) is that the distinction emerges between bulk-ZnO (“negligible solubility”) and nano-ZnO (“low solubility”) at neutral pH and between bulk-Zn metal (“moderate solubility”) and nano-Zn metal (“high solubility”) at low pH.

**Table 4:** Placing solubility results from Table 2 into different grouping schemes shows the importance of specifying pH

4a		
Test material	Low pH	Neutral pH
Bulk-Zn metal	Biopersistent	Biopersistent
Nano-Zn metal	Soluble	Biopersistent
Bulk-ZnO		
Nano-ZnO_100 nm_	Soluble	Biopersistent
Nano-ZnO_50 nm_		
Bulk-anatase		
Nano-anatase	Biopersistent	Biopersistent
Bulk-rutile		
Nano-rutile		
4b		
Solubility category	Low pH	Neutral pH
High solubility (>70 %)	Bulk-ZnO (88.5 %)	
	Nano-ZnO_100 nm_ (97 %)	
	Nano-ZnO_50 nm_ (94 %)	
	Nano-Zn metal (80.8 %)	
Moderate solubility (10–70 %)	Bulk-Zn metal (17.5 %)	
Low solubility (1–10 %)		Nano-Zn metal (1.33 %)
		Bulk-Zn metal (2.05 %)
		Nano-ZnO_50 nm_ (2.13 %)
		Nano-ZnO_100 nm_ (1.87 %)
Negligible solubility (<1 %)		Bulk-ZnO (0.95 %)
	Bulk-anatase (0.019 %)	Bulk-anatase (<0.0001 %)
	Nano-anatase (0.022 %)	Nano-anatase (<0.0001 %)
	Bulk-rutile (0.0001 %)	Bulk-rutile (<0.0001 %)
	Nano-rutile (0.0002 %)	Nano-rutile (<0.0001 %)

Table 4a (top) uses 100 mg/L as the screening criterion; Table 4b (bottom) uses four categories of water solubility based on percentage (%*M*_d_/*M*_o_)

In the context of read-across, the experimental results showed that using solubility data for bulk-ZnO as a substitute for nano-specific data actually yielded more conservative estimates of biopersistence at both ends of the pH spectrum (Table 3). With respect to TiO_2_, the dissolution kinetics of the bulk analogue yielded a more conservative estimate of biopersistence at low pH conditions, while at neutral pH, no significant difference could be discerned between the TiO_2_ nanomaterials and their bulk analogues (Table 3). The 2-h solubility assay yielded a similar set of observations: all four bulk analogues (including Zn metal, ZnO and two crystal forms of TiO_2_) yielded more conservative results for biopersistence at both pH conditions (Table 2).

## Conclusions

Particle size and crystal form of inorganic ENMs are both important properties that influence dissolution behaviour. However, in the context of grouping ENMs using solubility criteria, pH of the medium emerged as the key parameter for the studied ENMs. This point was demonstrated by the Zn compounds, all of which classified as biopersistent at neutral pH but soluble at low pH. These results pointed to limitations of using standard references on aqueous solubility such as the CRC handbook, which generally reports solubility at ambient temperature (not body temperature) and provides only qualitative information on the pH of the medium. The assays used in the present study were more applicable to human health risk assessment in that they assessed dissolution of ENMs at body temperature (37 °C) using two pH conditions (1.5 and 7) that approximately frame the pH range found in human body fluids.

Utembe et al. (2015) and others recommend dissolution kinetics for understanding biodurability of nanomaterials. However, in the context of preliminary screening assessments (tier 1), the results of this study showed few practical advantages to undertaking the difficult time series experiments necessary to derive rate constants and half-lives. The main benefit in the present study was a slight improvement in defining the effect of particle size on solubility. Time series experiments showed that the dissolution rates of ZnO materials increased (and half-lives decreased) with decreasing particle size in the following order: bulk-ZnO < nano-ZnO_100 nm_ < nano-ZnO_50 nm_ at both pHs (Table 3). This was an improvement in resolution compared to the 2-h solubility assay at low pH, which yielded inconsistent results for nano-ZnO_50 nm_ (Table 2), but this nanomaterial was the exception. Compared to the time series experiments, the 2-h assay was equally capable of distinguishing between dissolution of nano-ZnO_100 nm_ and its bulk-ZnO analogue at low pH and equally capable of displaying the bulk-ZnO < nano-ZnO_100 nm_ < nano-ZnO_50 nm_ dissolution trend at neutral pH (Table 2).

The main implication of this study is that pH and temperature should be specified for solubility testing, even at the preliminary (tier 1) screening level. In this study, initial concentration was kept constant for the purpose of investigating other parameters, but initial concentration is another parameter that should be considered as it can affect the apparent equilibrium concentration (Misra et al. 2012). More robust levels of risk assessment (beyond preliminary screening) typically require characterization of dissolution behaviour in whichever exposure medium is being used for toxicity testing, or in complex media that simulate various biological compartments, depending on the route of exposure (ECHA 2016; Arts et al. 2016). The potential disadvantage of adding other constituents to screening level assays is that these constituents may either enhance or decrease dissolution of ENMs due to interaction with dissolved ions (Li et al. 2011; Mu et al. 2014) and may induce agglomeration of ENMs (David et al. 2012; Mudunkotuwa et al. 2012). For example it may be advantageous to add NaCl to the dissolution medium to mimic the ionic strength of body fluids, but the presence of the Cl^−^ ligand may inadvertently impact the dissolution rate, either negatively or positively depending on the ENM being studied. As the next stage of this research, the impact of adding potentially interfering constituents to the dissolution medium of a standardized test will be investigated on a case-by-case basis.

## Electronic supplementary material

ESM 1 (PDF 311 kb)
