# Supplementary material for: Influence of pH, particle size and crystal form on dissolution behaviour of engineered nanomaterials
Source: Environ Sci Pollut Res Int. 2016 Oct 26;24(2):1553–64. doi: 10.1007/s11356-016-7932-2 (PMC5306302; doi:10.1007/s11356-016-7932-2)
Supplement: Supplementary file 1 — (PDF 311 kb) [file 11356_2016_7932_MOESM1_ESM.pdf]

Supplementary Material

M.-L. Avramescu, P.E. Rasmussen*, M. Chénier, and H.D. Gardner. Influence of pH, particle size and crystal form on dissolution behaviour of engineered nanomaterials. Environmental Science and Pollution Research. *email: Pat.Rasmussen@canada.ca

Characteristics of Nanomaterials and their bulk analogues used in this study

Powdered X-ray diffraction (XRD) and small-angle X-ray scattering (SAXS) were used to determine crystallographic structure and confirm identity purity of all materials used in the study (see Figures S1 for all XRD patterns). XRD and SAXS analyses were carried out at University of Ottawa X-ray facility using a Rigaku Ultima IV difractrometer. Powdered XRD pattern were collected using a Cu target with an acceleration voltage of 40 kV and a tube current of 40 mA at 2theta angles. For the SAXS the configuration of the Rigaku Ultima IV was changed to transmit X-rays through the sample. The powdered X-ray technique used in this study measures nanocrystal size, whereas the SAXS technique measures nanoparticle size and size distributions (from 1 to 65 nm) in powders and bulk samples. Rigaku's Nanosolver software (version 3.5) was used for the SAXS calculations for particle size. A spherical particle size was assumed for the calculated distribution model. It was assumed that purchased material used in this study had a homogeneous particle size. Both X-ray diffraction and SAXS provide results as a volume average diameter. Scherrer model were used to calculate nanocrystal size (average volume diameter) from samples analysed by powdered X-ray diffraction. All ZnO and TiO_2 samples were analyzed for sample purity and particle size and distinguish broadening associated with crystal size from broadening associated with crystal strain. A summary of the Scherrer/Williamson-Hall nanocrystal diameter estimates for all ZnO and TiO_2 samples is presented in Table 1.

Analysis of all samples by powdered XRD confirmed that all ZnO samples used in this study were in the wurzite form (Figure S1). The ZnO (101) diffraction peak is used to show differences in peak shape for the ZnO samples; the broadening of peaks associated with smaller particle size (Figure S1a insert). The broadest peak relative to peak height (i.e. smallest particles) is associated with nano-ZnO_{50nm} and the narrowest peak relative to peak height (i.e. largest particles) is associated with the bulk-ZnO. The calculated particle size by SAXS for nano-ZnO_{100nm} was 36 nm.

Anatase was the only mineral identified from the powdered diffraction patterns for both nano-$anatase_{25nm}$ and bulk-anatase samples. In contrast, both rutile samples (nano and bulk) were not pure, and anatase was identified as a minor constituent, less than 4% by Rietvald analysis (Table 1). The correlation between peak broadening and particle size for both anatase and rutile is shown in Figure S1b. As expected, the narrowest peak for rutile samples (Figure S1b) is associated with the bulk sample and the broadest peak is associated with NIST 1898 (size 37 ± 6 nm). Similarly for anatase samples (Figure S1b), the narrowest peak is associated with the bulk sample and the broadest peak is associated with the nano-$anatase_{25nm}$.

Generally, the Scherrer calculations for nanocrystal diameter and SAXS estimates for nanoparticle size agreed with the manufacturer claims.

Supplementary Material

M.-L. Avramescu, P.E. Rasmussen*, M. Chénier, and H.D. Gardner. Influence of pH, particle size and crystal form on dissolution behaviour of engineered nanomaterials. Environmental Science and Pollution Research. *email: Pat.Rasmussen@canada.ca

(a)

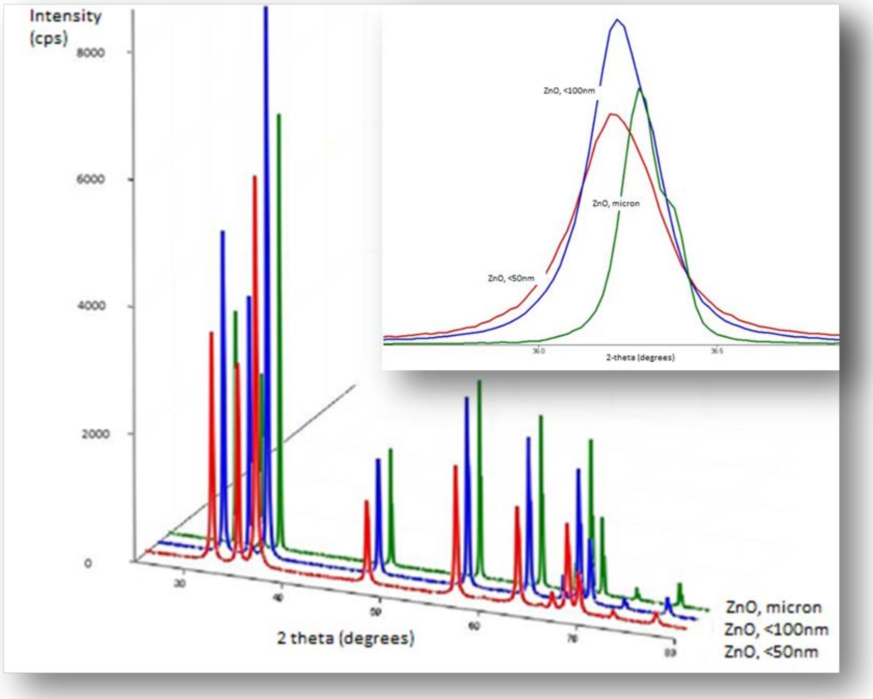

(b)

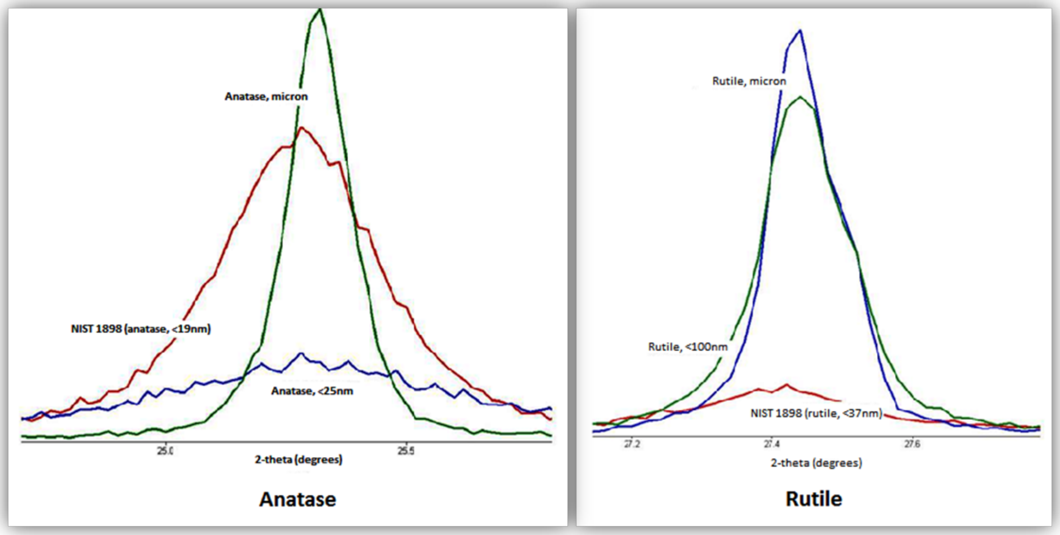

Figure S1 (a) Powdered X-ray diffraction patterns confirmed wurzite form for all ZnO samples; ZnO (1,0,1) diffraction peaks (insert).
(b) Anatase (1,0,1) and Rutile (1,1,0) diffraction peaks showing peak broadening with smaller particle size in ENMs (Note: determined crystal size for nano-anatase$_{25nm}$ is 7nm).

M.-L. Avramescu, P.E. Rasmussen*, M. Chénier, and H.D. Gardner. Influence of pH, particle size and crystal form on dissolution behaviour of engineered nanomaterials. Environmental Science and Pollution Research. *email: Pat.Rasmussen@canada.ca

Solubility 2hr assay comparison separation methods

Table S1 shows the comparison of the dissolution results using syringe filtration versus centrifugation presented as concentration (mg/L or µg/L) of nano and bulk ZnO and TiO_2 samples at low pH (1.5) and neutral pH (7) using the short (2hr) assay. For ZnO samples no significant difference ($p>0.05$) was observed at low pH between the dissolved Zn concentrations measured after centrifugation and syringe filtration regardless of the size. In contrast, at neutral pH dissolved Zn concentrations obtained after centrifugation where significantly higher ($p<0.05$) than those obtained after syringe filtration (Table S1). This was also observed for the TiO_2 samples at low and high pH, where dissolved Ti concentrations obtained after centrifugation were significantly higher ($p<0.05$) than those obtained after syringe filtration (Table S1). These results show that centrifugation is not able to fully separate remaining particles from dissolved fraction and demonstrate the need for more accurate separation techniques. Although optimization of the 2 hr assay (by increasing the speed and time of centrifugation) may give acceptable results for separation of dissolved metal and nanoparticles as observed previously (Li et al. 2011, Xia et al. 2008, Xiao et al. 2015) centrifugation is not suitable our for time series experiments.

Table S1 Comparison of the dissolution results using syringe filtration versus centrifugation presented as concentration (mg/L or µg/L) dissolved of nano and bulk ZnO and TiO_2 samples at low pH (1.5) and neutral pH (pH 7) estimated with the short (2hr) assay. Data are presented as mean and standard deviation of five independent replicates.

Description	Units	low pH		neutral pH	
		Syringe filtration	Centrifugation	Syringe filtration	Centrifugation
nano-ZnO_{50nm}	mg/L	391.3 ± 5.11	393.9 ± 4.53	7.85 ± 0.27	11.7 ± 0.74
nano-ZnO_{100nm}	mg/L	379 ± 5.25	393.1 ± 17.7	8.59 ± 0.45	[a]27.4 ± 3.32
bulk-ZnO	mg/L	368.7 ± 15.3	370.5 ± 19.2	4.12 ± 0.21	6.00 ± 0.51
nano-$Rutile_{100nm}$	ug/L	0.505 ± 0.059	56.5 ± 2.5	0.210 ± 0.058	76.2 ± 40.5
bulk-Rutile	ug/L	0.282 ± 0.032	43.0 ± 13.0	0.146 ± 0.069	32.3 ± 8.87
nano-$Anatase_{25nm}$	ug/L	69.3 ± 6.44	734.2 ± 382.1	0.197 ± 0.069	2199 ± 941.1
bulk-Anatase	ug/L	58.5 ± 6.08	395.2 ± 190.5	0.190 ± 0.075	1793 ± 1451.5
[b]Nist 1898	ug/L	39.1 ± 3.65	1070 ± 732.1	0.128 ± 0.040	3258 ± 856.3

[a]extract centrifuged two times; [b]n=3

M.-L. Avramescu, P.E. Rasmussen*, M. Chénier, and H.D. Gardner. Influence of pH, particle size and crystal form on dissolution behaviour of engineered nanomaterials. Environmental Science and Pollution Research. *email: Pat.Rasmussen@canada.ca

Explanation for Footnote 1, Table 3. Bulk-ZnO dissolution data were fitted with Eq. 2a (two negative exponentials) corresponding to biphasic dissolution behaviour. The fraction of the bulk-ZnO dissolved in the initial phase was less than 2% (f =1.24 %, k = 42.9 d^{-1}; $t_{1/2}$ = 0.016 d, and k_{SSA} = 3.41 x 10^-4 g/(cm^2d)) and consequently, the reported dissolution rate corresponds to the long-term phase.

Explanation for Footnote 2, Table 3. For nano-anatase the model did not fit well using the complete set of time series results (r^2=0.34, p=0.15), and fit very well using only the initial first 60min data (r^2=0.69, p=0.0001). Considering that at low pH the nano anatase sample released the most Ti in the first 60min, the kinetic parameters estimated from these initial data (first 60 min) are presented for comparison.

Increased release of Ti in the initial stage (first 60 min) of dissolution was observed in our time series experiments for nano-anatase at low pH. This is in agreement with Schmidt and Vogelsberger (Schmidt and Vogelsberger 2006) who reported the appearance of a "*steep increase of the solubility at the beginning of dissolution*" process followed by a "*relatively constant value thereafter for TiO_2 nanomaterials*"(at pH 1.5; 0.1M NaCl, 25 and 37°C). The authors (Schmidt and Vogelsberger 2006) stress the importance of measuring the dissolution kinetics "*in the very first stage of the process*" and recognize the difficulties due to the time required by certain procedural steps as sampling, filtration, etc. A comprehensive interpretation of this observed effect is presented by Schmidt and Vogelsberger (Schmidt and Vogelsberger 2009) based on a thermodynamic and kinetic effect. Considering those, for nano-anatase sample (crystal size 7nm) the parameters estimated with the initial data (0-60min) fit were used for comparison (Table 3). The results for the fitted equation parameters estimated with all data (0-240min) were also presented as footnote (k= 0.47 x 10^-3 d^{-1}; $t_{1/2}$ = 14.6 x 10^2 d, and $k_{SSA=}$ 0.86 x 10^-9 g/(cm^2d), r^2=0.34, p=0.15) in Table 3 for information purpose only.

Moreover, the parameters estimated with the initial data (0-60min) fit for the bulk-anatase sample are: k= 3.07 ± 0.28 x 10^-3 d^{-1}; $t_{1/2}$ = 226 d, and $k_{SSA=}$ 13.6 x 10^-9 g/(cm^2d) , r^2=0.90, p<0.00001. Those results also show that the nano-anatase sample (k=3.26 x 10-3 d^{-1}, $t_{1/2}$ = 213 d, Table 3) dissolve faster than bulk anatase sample (k=3.07 x 10-3 d^{-1}, $t_{1/2}$ = 226 d).

References

Li M, Zhu L, Lin D (2011) Toxicity of ZnO nanoparticles to escherichia Coli: Mechanism and the influence of medium components. Environmental Science and Technology 45:1977-1983

Schmidt J, Vogelsberger W (2009) Aqueous long-term solubility of titania nanoparticles and titanium(IV) hydrolysis in a sodium chloride system studied by adsorptive stripping voltammetry. Journal of Solution Chemistry 38:1267-1282

Schmidt J, Vogelsberger W (2006) Dissolution kinetics of titanium dioxide nanoparticles: The observation of an unusual kinetic size effect. J Phys Chem B 110:3955-3963

Xia T, Kovochich M, Liong M, Mädler L, Gilbert B, Shi H, Yeh JI, Zink JI, Nel AE (2008) Comparison of the mechanism of toxicity of zinc oxide and cerium oxide nanoparticles based on dissolution and oxidative stress properties. ACS Nano 2:2121-2134

Xiao Y, Vijver MG, Chen G, Peijnenburg WJGM (2015) Toxicity and accumulation of Cu and ZnO nanoparticles in daphnia magna. Environ Sci Technol 49:4657-4664
